# Native Mass Spectrometry Captures the Conformational Plasticity of Proteins with Low-Complexity Domains

**DOI:** 10.1021/jacsau.4c00961

**Published:** 2025-01-08

**Authors:** Hannah Osterholz, Alexander Stevens, Mia L. Abramsson, Dilraj Lama, Klaus Brackmann, Anna Rising, Arne Elofsson, Erik G. Marklund, Sebastian Deindl, Axel Leppert, Michael Landreh

**Affiliations:** † Department of Cell and Molecular Biology, Uppsala University, 751 24 Uppsala, Sweden; ‡ Department of Microbiology, Tumor and Cell Biology, Karolinska Institutet, 171 65 Solna, Sweden; § Department of Animal Biosciences, Swedish University of Agricultural Sciences, 750 07 Uppsala, Sweden; ∥ Department of Medicine Huddinge, Karolinska Institutet, 141 83 Huddinge, Sweden; ⊥ Department of Biochemistry and Biophysics and Science for Life Laboratory, Stockholm University, 171 21 Solna, Sweden; # Department of Chemistry-BMC, Uppsala University, 751 23 Uppsala, Sweden; ¶ Department of Cell and Molecular Biology, Science for Life Laboratory, Uppsala University, 751 24 Uppsala, Sweden

**Keywords:** intrinsic disorder, electrospray ionization, protein engineering, liquid−liquid phase separation

## Abstract

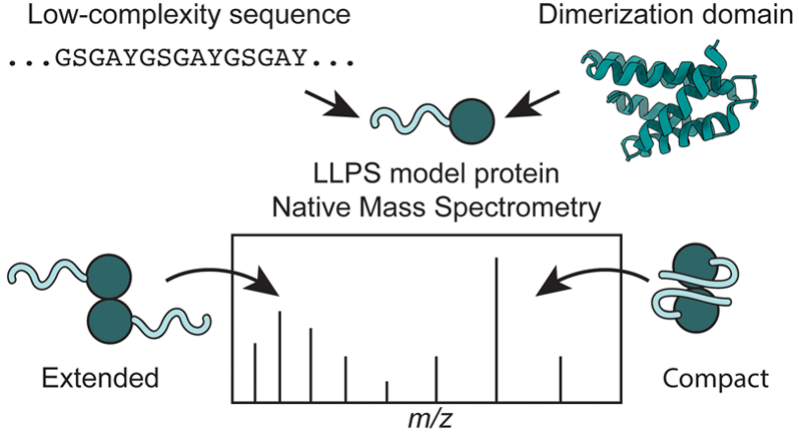

Disordered regions are an important functional feature of many multidomain proteins. A prime example is proteins in membraneless organelles, which contain folded domains that engage in specific interactions and disordered low-complexity (LC) domains that mediate liquid–liquid phase separation. Studying these complex architectures remains challenging due to their conformational variability. Native mass spectrometry (nMS) is routinely employed to analyze conformations and interactions of folded or disordered proteins; however, its ability to analyze proteins with disordered LC domains has not been investigated. Here, we analyze the ionization and conformational states of designed model proteins that recapitulate key features of proteins found in membraneless organelles. Our results show that charge state distributions (CSDs) in nMS reflect partial disorder regardless of the protein sequence, providing insights into their conformational plasticity and interactions. By applying the same CSD analysis to a spider silk protein fragment, we find that interactions between folded domains that trigger silk assembly simultaneously induce conformational changes in the LC domains. Lastly, using intact nucleosomes, we demonstrate that CSDs are a good predictor for the disorder content of complex native assemblies. We conclude that nMS reliably informs about the conformational landscape of proteins with LC domains, which is crucial for understanding protein condensates in cellular environments.

## Introduction

Intrinsically disordered proteins (IDPs) fulfill a plethora of biological functions, using their conformational promiscuity to mediate an array of interactions.^1^ However, most disordered sequences in the proteome are part of multidomain proteins that combine folded and disordered regions in the same polypeptide chain.^2,3^ Prominent examples of such mixed-structure proteins are found in membraneless organelles, cellular superstructures that form via liquid–liquid phase separation (LLPS).^4^ Proteins in membraneless organelles often contain disordered low-complexity (LC) sequences that engage in weak, nonspecific interactions, but also folded domains that perform specific biological functions.^5^ Probing the conformational landscape and interactions of these proteins is challenging, as the structural dynamics of their domains occur on vastly different time scales and are strongly affected by the complex environment, for example, inside liquid condensates.

Native mass spectrometry (nMS) with nanoelectrospray ionization (nESI) allows the analysis of protein interactions and conformations by transferring intact protein complexes from solution to the gas phase. nMS is predominantly applied to stably folded proteins that experience comparatively little distortions during desolvation and (positive) ionization.^6^ Folded proteins ionize via the charge residue model (CRM).^7,8^ According to the CRM, the solvent surrounding the protein slowly evaporates to dryness during transfer to vacuum, giving rise to compact ions with narrow charge state distributions (CSDs).^9,10^ Importantly, the total ion charge is determined by the solvent-accessible surface area (SASA) of the protein and is independent of the number of ionizable residues.^11^ Unfolded proteins, on the other hand, can ionize via the chain ejection mechanism (CEM).^7,12^ Here, disordered proteins are preferentially located at the surface of electrospray droplets, where arginine and lysine residues become protonated. The protein is ejected from the droplet through charge repulsion between the ionizable residues and the droplet surface, which results in broad CSDs and a maximum charge state close to the number of basic (or acidic) residues.^13,14^ IDPs can ionize via both mechanisms, producing multimodal CSDs that range from compact, lowly charged to unfolded, highly charged ions.^15^ Ion mobility measurements have suggested that the gas-phase conformations of IDPs are strongly affected by the charge state, experiencing Coulombic stretching and collapse at high and low charges, respectively, which can differ significantly from their *R*_g_ in solution.^16,17^ However, the distribution between high and low charge states appears to reflect the distribution of extended and compact conformations in solution.^15,18^ The ability of nMS to capture the conformational preferences of IDPs has been attributed to the presence of ionizable residues. For example, the CSDs of IDPs can be modulated by changing the location of ionizable residues.^19^ Beveridge, Barran, and co-workers have utilized a combination of nMS and ion mobility spectrometry to assess the extent of disorder in protein ions. Their framework suggests that proteins that contain both folded and disordered domains will use a combination of both ionization mechanisms, leading to a low-intensity population of high-charge states and a high-intensity, narrow population of low-charge states.^20^

While the ionization and gas-phase behavior of completely disordered proteins is relatively well understood,^15−17^ several recent studies have turned to nMS to capture interactions of phase-separating proteins that are partially disordered and contain large LC domains. Examples include the stress granule proteins FUS and TDP-43, nucleolar scaffold protein NPM1, spider silk proteins (spidroins), and ubiquitin-binding proteins.^21−24^ While analysis of folded and disordered proteins with nMS thus builds on a good understanding of CRM and CEM, it is not clear how phase-separating proteins fit into the framework, as they:

(a) contain folded and disordered domains in the same chain,
(b) contain LC sequences that can be entirely chargeless or entirely composed of charged residues, and
(c) simultaneously engage in nonspecific and specific interactions.

**Figure 1:**
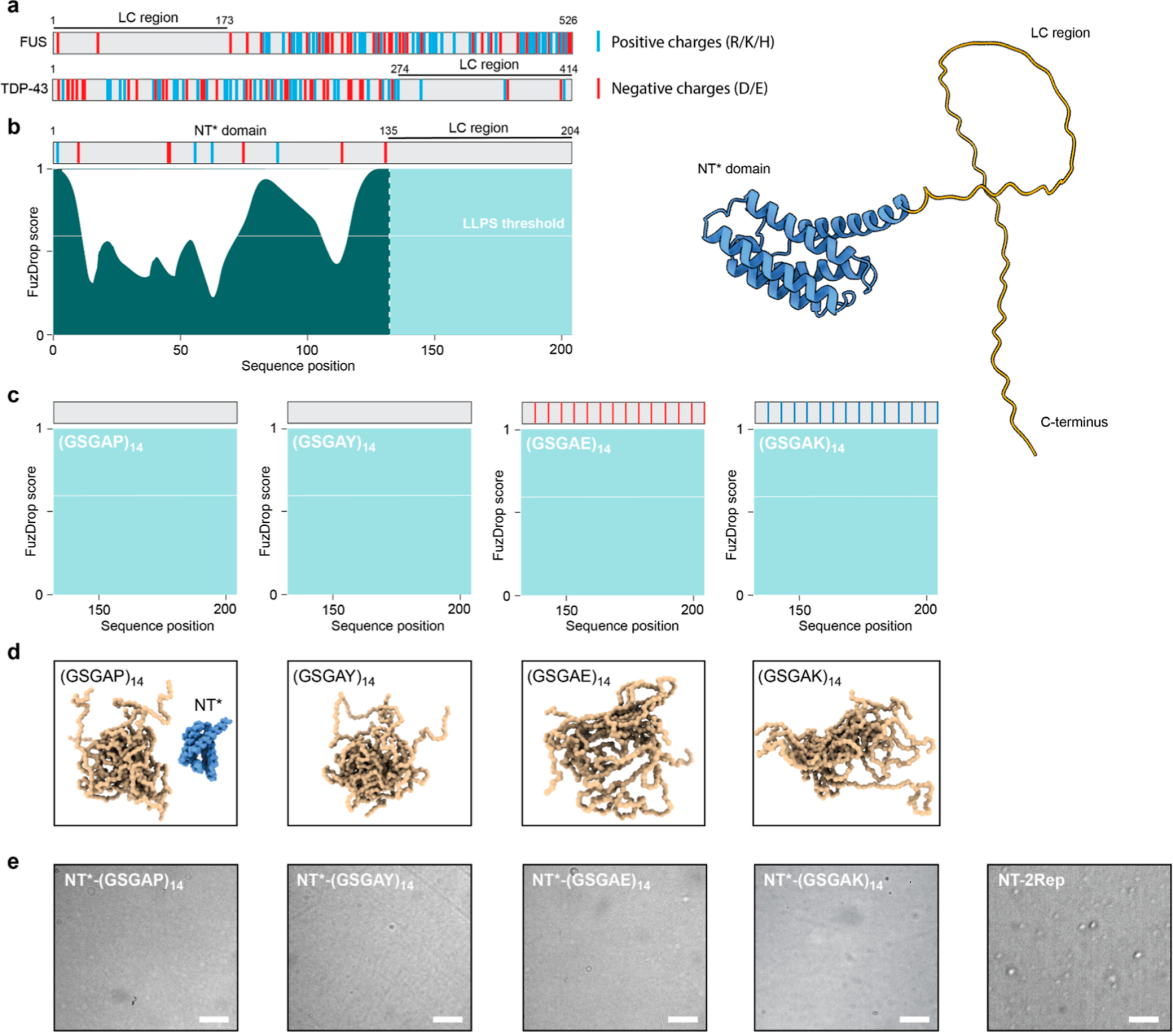
Design of partially folded proteins with disordered LC domains. (a) FUS and TDP-43 are examples of proteins that undergo LLPS via LC domains that are virtually devoid of charged residues. (b) Overall architecture of the model proteins shown as FuzDrop score (left) and AF3 model (right). The NT* domain (teal) scores low for LLPS propensity and is connected to a 70-residue LC domain with high LLPS propensity (mint). The locations of residues with positive (blue) and negative (red) solution charge are indicated in the gray bars above the FuzDrop plots. (c) FuzDrop scores of the (GSGAP)_14_, (GSGAY)_14_, (GSGAE)_14_, and (GSGAK)_14_ repeats show high LLPS propensity. (d) IDPGan ensembles of ten copies of each repeat show complete disorder and a slightly lower degree of compaction for the (GSGAE)_14_ and (GSGAK)_14_ repeats. The NT* domain (blue) is shown for scale in the left panel. (e) Light microscopy images of 20 μM NT*-LC proteins in 100 mM ammonium acetate, pH 8, show no droplet formation. NT-2Rep droplets formed in 0.75 M phosphate buffer are shown as positive control on the far right. Scale bars are 10 μm.

For example, FUS and TDP-43 contain LC sequences that mediate LLPS and are free of charges despite being >150 residues long (Figure 1a), but also include folded RNA-binding domains, and, in the case of TDP-43, a folded N-terminal oligomerization domain.^25−28^ The possibility to study the structures and interactions of phase-separating proteins with nMS thus raises three fundamental questions: (i) can we detect partial disorder with nMS? (ii) does the amino acid composition of the LC domains affect ionization? (iii) can nMS capture conformational changes in the LC domains?

Here, we address these questions using model proteins that combine a folded domain with disordered regions that resemble the LC domains found in proteins that undergo LLPS. We find that the CSDs of these proteins reflect their relative disorder content. In the absence of positively charged residues, the CSDs appear to be dictated by SASA, using instead ammonium ions as charge carriers. Using a spidroin fragment with a folded domain and two LC repeats, we demonstrate that nMS captures conformational changes in the LC domain induced by dimerization of the folded domains. Lastly, we use CSDs in nMS to quantify histone tail disorder in intact nucleosomes and find good agreement with high-resolution structures. We conclude that with nMS, we can explore the conformational space of proteins with disordered LC domains, which opens new avenues for the analysis of protein condensates.

## Results

### Design of Proteins with Disordered LC Domains

To investigate the relationship among disorder, amino acid composition, and ionization of proteins with LC domains, we designed a group of model proteins with a folded domain and a disordered LC domain. As a starting point, we chose major ampullate spidroin 1, the main component of spider dragline silk, which has a folded N-terminal (NT) domain followed by a repetitive low-complexity domain composed of alternating poly-A and G/S/Y-rich blocks.^29^ In native spidroins, the NT domain undergoes pH-dependent dimerization, and the repeats in the LC domain self-assemble.^22,30,31^ We abolished NT-dimerization by introducing the D42K/K65D mutation (NT*)^32^ and replaced the LC domain with designed 14 penta-repeats of either GSGA(P/Y/K/E) to reflect the intrinsic disorder and narrow amino acid composition of LC domains. Glycine (G), serine (S), and alanine (A) are commonly enriched in LC domains of phase-separating proteins,^33−35^ and proline (P) is associated with disorder. Tyrosine (Y) and lysine (K) engage in weak π–π- and π–cation interactions. In this manner, they mediate contacts between phase-separating protein assemblies, representing the “stickers” in the “stickers and spacers” model of LLPS,^36,37^ and the negatively charged glutamic acid (E) is found in LC domains of proteins that phase-separate together with RNA. In eukaryotic proteins, individual LC domains are on average 42 residues long but can reach up to several hundred residues in length.^38^

To assess whether the artificial sequences recapitulate key features of LC domains in LLPS-forming proteins, we turned to the AlphaFold3 (AF3)^39^ and the FuzDrop server^40^ (Figure 1b). Computational models suggest that the conformations of disordered domains that lack charged or hydrophobic residues are unaffected by the presence of folded domains in the same polypeptide chain. AF3 predictions suggest that the NT* domain retains its native fold in all variants, while the LC region is disordered (Figure 1b). The FuzDrop server predicts the propensity of proteins to phase-separate based on the occurrence of LC sequences. As expected, all four variants exhibit the maximum score for LLPS (Figure 1c). We additionally analyzed ensembles of the LC domains with IDPGan,^41^ which uses machine learning to generate representative conformational ensembles of IDPs. The (GSGAP)_14_, (GSGAY)_14_, (GSGAE)_14_, and (GSGAK)_14_ domains are completely disordered, with the (GSGAE)_14_ and (GSGAK)_14_ domains exhibiting slightly more dispersed ensembles due to intramolecular charge repulsion (Figure 1d).

However, we also needed to avoid assembly of the proteins into droplets under nMS conditions. Droplet formation would potentially bias the structural information that can be obtained since nMS captures predominantly soluble species. We, therefore, kept the LC domains to half the length of the naturally occurring LC domains in FUS and TDP-43, which, we reasoned, should reduce their LLPS propensity in most buffers. Light microscopy imaging of the four proteins in 100 mM ammonium acetate, pH 8, which is a standard nMS buffer and does not prohibit droplet formation,^21,22,24^ confirmed the absence of any droplets (Figure 1e). Based on these considerations, we conclude that our model system recapitulates features of LLPS-associated proteins with folded domains and LC sequences but retains sufficient solubility to facilitate nMS analysis.

### Partial Disorder Is Partially Retained in nESI

**Figure 2:**
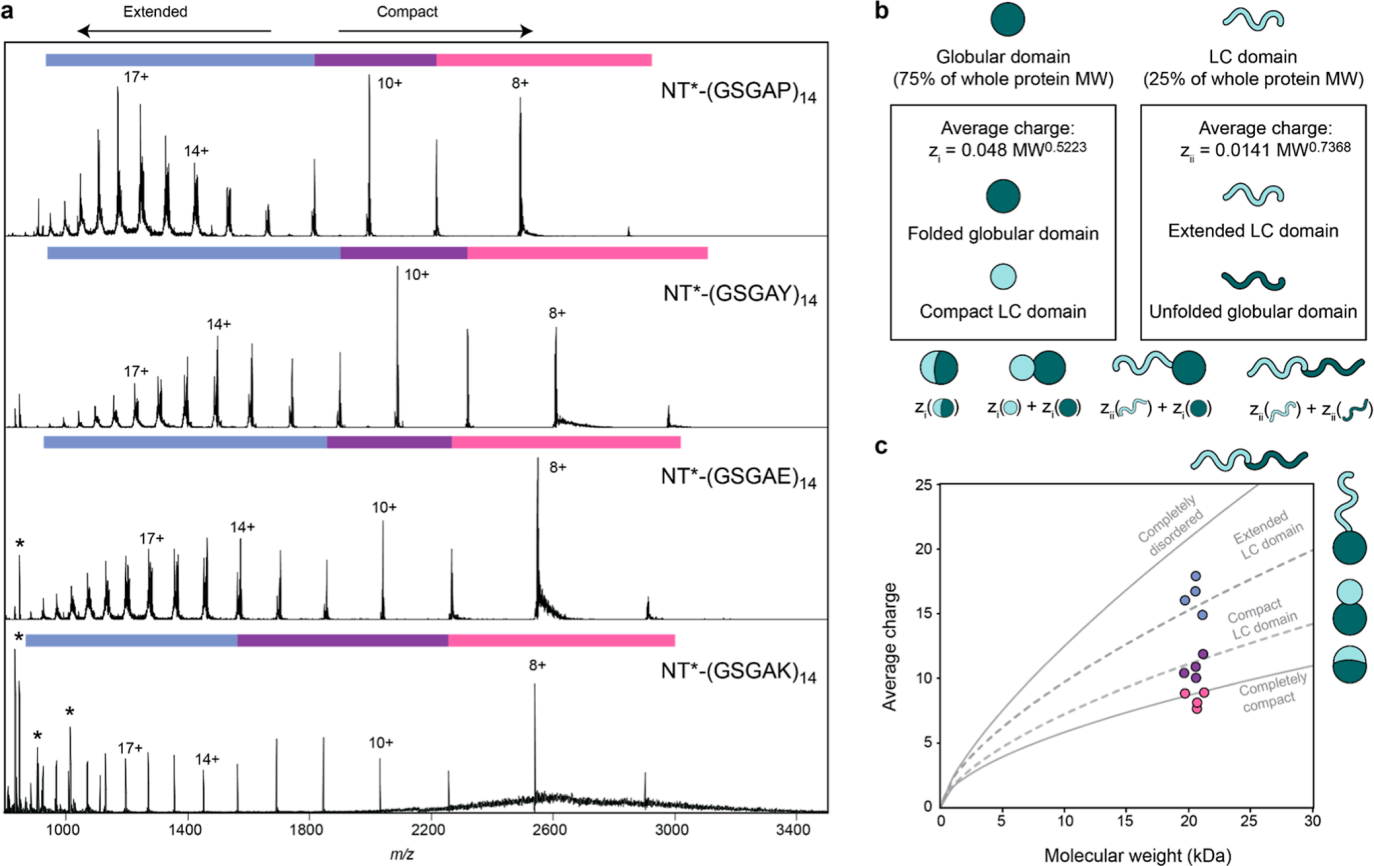
Charge states of partially folded model proteins with LC domains reflect relative disorder content and surface area. (a) Representative native mass spectra for NT*-(GSGAP)_14_, NT*-(GSGAY)_14_, NT*-(GSGAE)_14_, and NT*-(GSGAK)_14_ (top to bottom) show trimodal CSDs. The highest charge state envelope is highlighted in blue and centered on 17+, 14+, 16+, and 17+, respectively, while the intermediate envelope (purple) and the lowest envelope (pink) are centered on 10+ and 8+. Asterisks indicate nonprotein peaks. (b) Predicting the charge of a protein with a globular (teal) and a disordered domain (mint). The mass of the disordered domain is 25% of that of the whole protein, which is the case for NT-LC proteins used here. The expected charge of each domain is calculated separately using the empirical formulas for either compact (i) or disordered proteins (ii). The expected charges are then summed to predict the total charge for a protein in which both domains are disordered, the LC domain is disordered, both domains are compacted separately, or both domains are compacted together. (c) Comparison of the predicted and experimental charge states for partially disordered proteins. Expected average charges as a function of molecular weight are shown as solid lines for fully disordered and for completely folded proteins. Dashed lines indicate the expected average charge for a protein with an extended disordered domain or a compact disordered domain. The average charges of the three CDSs for each of the NT*-LC proteins are shown using the same color code as in (a) and correspond to a compact and an unfolded domain (blue), two compact domains (purple), and a single collapsed protein (pink).

As the first step, we recorded nMS data for the four model proteins (Figure 2a). Mass spectra obtained from 100 mM ammonium acetate, pH 8, show charges from 7+ to 22+ and three CSDs. The spectra were found to be highly reproducible between protein batches. The two lower CSDs are centered on the 8+ and 10+ charge states for all proteins, and the highest CSDs are centered on the 17+, 14+, 16+, and 17+ charges for NT*-(GSGAP)_14_, NT*-(GSGAY)_14_, NT*-(GSGAE)_14_, and NT*-(GSGAK)_14_, respectively. Disordered proteins generally ionize with a wider range of charge states than compact proteins and often show multiple CSDs that can be attributed to the protein existing in various conformations.^9,20,42^ Since our model proteins contain both a structured and a disordered part, we asked how the CSDs correlate with the expected folded states. We plotted the expected average charge as a function of molecular weight for completely folded and completely disordered proteins from empirical correlations (Figure 2b).^43−45^ The average charges of the lowest CSD for the four proteins (pink) are between 8.3 and 8.4 and agree well with the expected charge for globular proteins of the same molecular weight, suggesting ionization according to the CRM (Figure 2c). The average charges of the highest CSDs for each protein (blue) are 16.3, 14.8, 16.5, and 17.8 for NT*-(GSGAP)_14_, NT*-(GSGAY)_14_, NT*-(GSGAE)_14_, and NT*-(GSGAK)_14_. However, for a completely disordered protein of the same molecular weight, the expected average charge is approximately 22+.

The fact that we observe notably lower average charges even for the highest CSD may indicate that the proteins are not fully disordered during ionization. As outlined above, the folded NT* domain accounts for ca. 75% of the protein’s mass and the disordered LC domain for 25%. We therefore combined the prediction of the average charge for folded and unfolded proteins by treating 25% of the proteins as disordered and 75% as folded and calculated the sum of the resulting charges (Figure 2b). We found the high CSDs (blue) to be in good agreement with this prediction, meaning they exhibit average charges expected for a protein that is 25% disordered (Figure 2c). We speculate that the highest charge state envelope arises from chain ejection of the LC domain from the ESI droplet, while the attached folded NT domain causes an overall lower total charge than expected for ionization via the CEM. Importantly, we cannot conclude from these data whether the NT domain is natively folded post-ionization. As for the intermediate CSD (purple), the average charge can be approximated by adding together the predicted average charges for the folded NT* domain and a compacted LC domain (Figure 2b,c). We speculate that the middle CSD may represent a partially folded state of the protein, which could arise through compaction in the shrinking ESI droplet in the absence of chain ejection, following an intermediate regime between CEM and CRM that was predicted previously for partially denatured proteins.^17^ We additionally investigated the effect of chemical denaturation with 5% formic acid on the CSDs of the variants (Figure S1). For NT*-(GSGAY)_14_, NT*-(GSGAP)_14_, and NT*-(GSGAE)_14_, the signal intensity of the population with the lowest charge is notably reduced, while the middle and highest charge states remain unaffected. NT*-(GSGAK)_14_ displays a higher maximum charge state than do the other variants, which is likely caused by the fully protonated LC domain. These findings agree with previous reports that proteins lacking ionizable residues do not exhibit broader CSDs or higher charge states in response to unfolding.^11^

### Charging of LC Domains Is Not Dependent on Ionizable Residues

**Figure 3:**
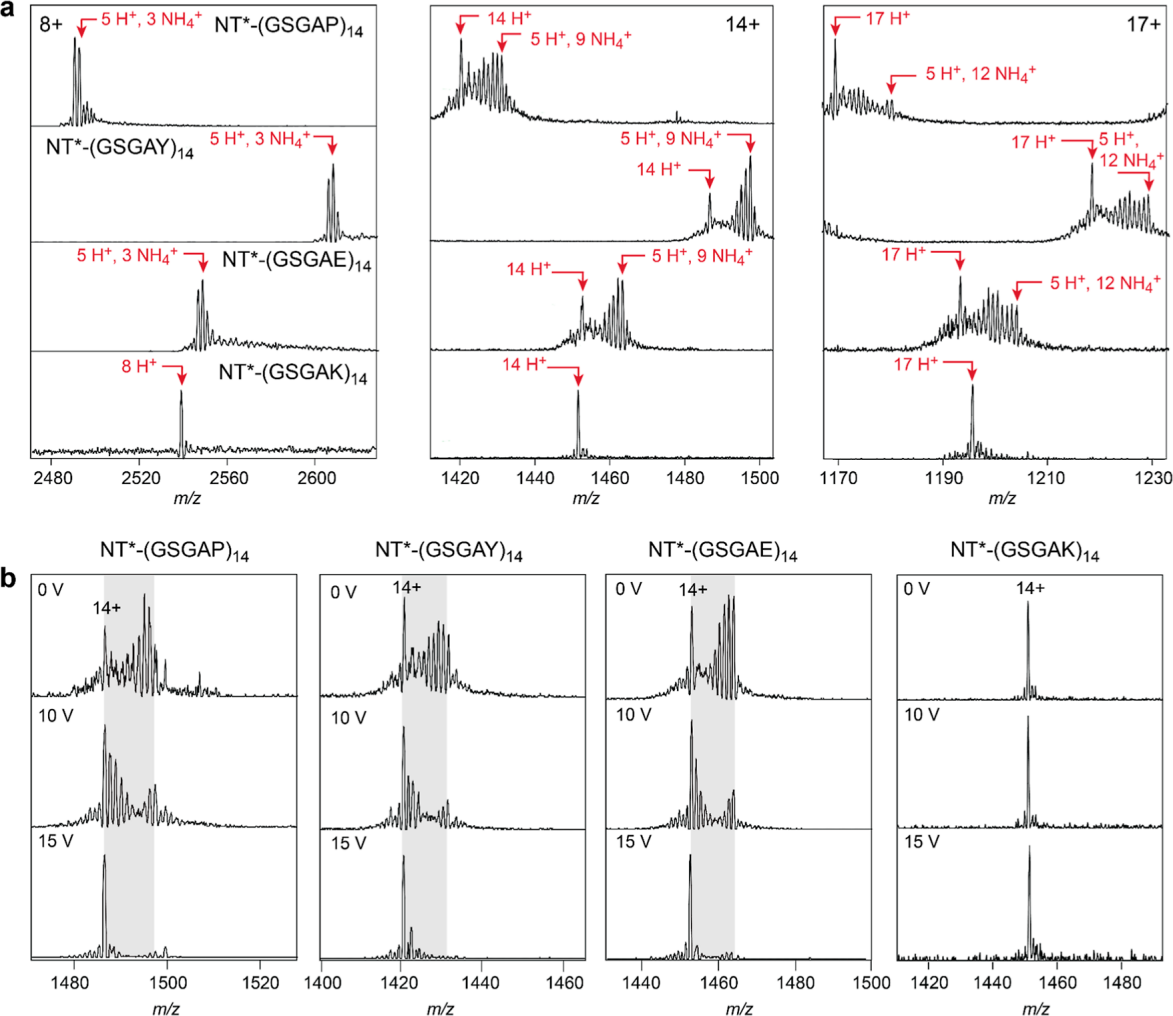
Disordered regions without positively charged residues ionize using NH_4_^+^ as charge carriers and retain protons upon collisional activation. (a) Representative native mass spectra of NT*-(GSGAP)_14_, NT*-(GSGAY)_14_, and NT*-(GSGAE)_14_ show a mixture of charge carriers. Each charge state has five protons and a variable number of ammonium ions. Higher charge states (>9+) also exist as an isolated peak with only protons as charge carriers. For NT*-(GSGAK)_14_, which contains an excess of residues with a positive solution charge, no ammonium adducts were observed for the same charge states. (b) Collisional activation results in the loss of ammonia adducts, leaving the proton-only peak.

The observation of CSDs associated with disorder suggests that the NT*-LC proteins may be able to ionize via the CEM. Previously, the ionization of charged residues exposed to the air–water interface has been suggested to drive the ejection of the disordered chain from the ESI droplet.^9,46^ However, the LC domains of three of our four proteins are devoid of positively charged residues. Upon closer examination of the individual charge states of these proteins, we detected several adduct peaks (Figure 3a). Here, the 8+ charge state shows a mass shift of 51 Da with two peaks separated by 17 Da. The 14+ charge states show a maximum mass shift of 153 Da and a 17+ charge state of 204 Da, both caused by multiple 17 Da adducts. The pattern, which extends to all charge states (Figure S2), suggests that five of the charges are protons, and the remaining charges are ammonium ions. Since NT* has five basic sites including the N-terminus, the protein thus likely ionizes these five sites and then uses ammonium as charge carrier to reach the charge dictated by the SASA. This model is corroborated by the fact that NT*-(GSGAK)_14_, which has 19 basic sites, does not retain any ammonium adducts (Figure 3a).

For higher charge states of NT*-(GSGAP)_14_, NT*-(GSGAY)_14_, and NT*-(GSGAE)_14_, we also observe a “proton-only” peak (Figure 3a). When the proteins were electrosprayed from dH_2_O in the absence of ammonium acetate, no proton-only peaks could be detected (Figure S2). Instead, the chargeless variants retained several Na^+^ adducts as charge carriers. Presumably, the high ammonium concentration in the previous experiments outcompeted Na^+^ so that the latter was unlikely to remain in most droplets in the late stages of ESI, whereas in dH_2_O, they make up a larger fraction of the charge carriers. To determine how protonation and ammonium adduction are related, we performed collisional activation of the ions. With increasing collision energy, ammonium adduct peaks diminish and the proton-charged peaks become more prominent (Figure 3b). However, the energy required to remove ammonium adducts decreases with an increasing charge state. At a collision voltage of 15 V, the ammonium adducts from the 14+ peak are almost completely removed, while adduct removal from the 8+ peak requires 25 V (Figure S2). We observe an activation-dependent charge reduction above 15 V. At this voltage, most of the ammonium adducts for higher charge states have already been dissociated, and the charge reduction coincides with the onset of fragmentation (Figure S2). These data indicate that a part of the ammonium adducts dissociates as ammonia, leaving behind a proton. Since proton transfer from ammonium ions to proteins is temperature-dependent,^47^ the increasing intensity of proton-only peaks for higher charge states could be explained by the higher activation experienced by these ions. Interestingly, the 8+ charge state of NT*-(GSGAE)_14_ retained ammonium adducts at collision voltages slightly higher than those of the other two variants (Figure S2).

We additionally tested the role of adducts by analyzing the proteins in negative ionization mode (Figure S3). We observed multiple 59 Da adducts on NT*-(GSGAP)_14_ ions, which is in line with bound acetate ions. The number of adducts correlated linearly with the charge state, with the 9– showing two adducts and the 10– having three adducts. NT*-(GSGAE)_14_ and NT*-(GSGAY)_14_ which contain an excess of negatively ionizable residues did not retain any adducts. No spectra could be obtained for NT*-(GSGAK)_14_. Considering that NT*-(GSGAY)_14_ and NT*-(GSGAP)_14_ each have seven sites that are negatively charged in solution, the data suggest that these proteins retain acetate ions as charge carriers for any additional negative charge, analogous to the retention of ammonium ions in positive ionization mode.

Together, these observations suggest that the adducts are attached to the LC domain. In positive ionization mode, in the absence of arginine and lysine side chains, ammonium ions attach to backbone carbonyl moieties, which have gas-phase basicities between 202 and 208 kcal/mol.^48^ This range is close to the gas-phase basicity of ammonia at 196 kcal/mol, which may explain why some ammonium ions remain bound to the chargeless LC domains instead of undergoing proton transfer. Konermann and colleagues have demonstrated that ammonium ions are not volatile per se but become volatile upon proton transfer to acetate or a protein.^49^ If proton transfer does not occur, for example, if the ion is attached to a backbone carbonyl with low gas-phase basicity, it is observed as a metastable adduct instead.

### nMS Reveals Compaction of an LC Region via Dimerization of a Folded Domain

**Figure 4:**
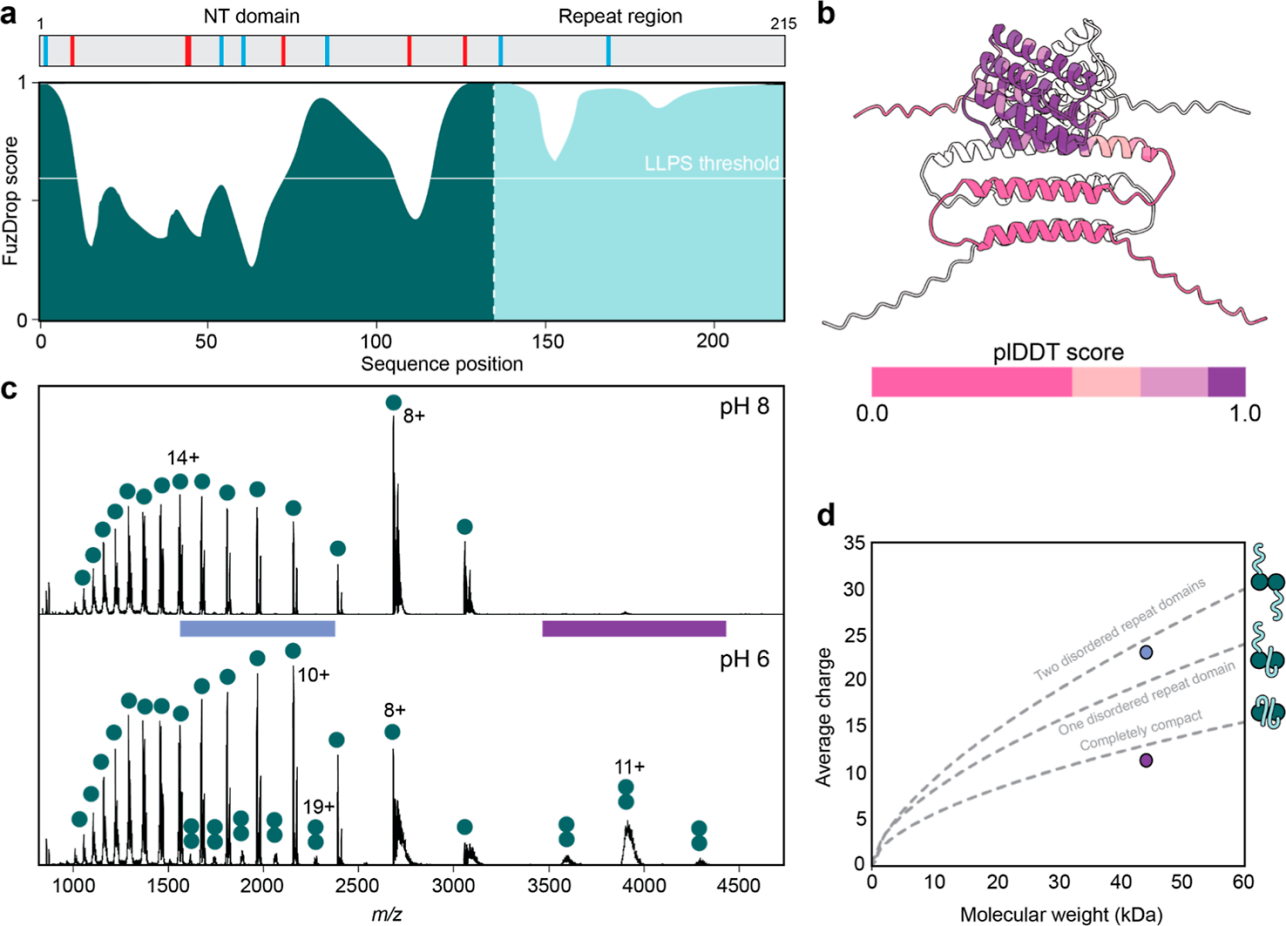
nMS captures conformational changes in the LC domain of a spider silk protein. (a) NT-2Rep fragment contains the wild-type NT domain and two LC repeats with seven basic residues in total. The LC domain is predicted to undergo LLPS. The gray stripe above the graph indicates the location of residues with positive (blue) and negative solution charge (red). (b) AF3 structure prediction of the dimerized truncated NT-2Rep version, with plDDT score coloring of one subunit and a transparent second subunit behind it. (c) Representative native mass spectra of NT-2Rep at pH 8 (top) and pH 6 (bottom) show a broad CSD. At low pH, two dimer populations can be observed that are centered on the 21+ (blue) and 9+ (purple) charge states. (d) CSD predictions for NT-2Rep dimers with both LC domains extended, one LC domain collapsed, and both LC domains collapsed show that the high and low experimental CSDs agree with two extended and two collapsed LC domains, respectively.

Next, we sought to apply the insights from the designed NT*-LC model proteins to investigate conformational changes in a naturally occurring low complexity domain. For this purpose, we turned to NT-2Rep, a truncated version of the MaSp1 spidroin that includes the WT folded NT domain and two of the LC repeats from the native MaSp1 protein (Figure 4a).^29^ WT NT is a monomer at pH 7 and dimerizes below pH 6.3.^50^ The LC region is capable of LLPS at high phosphate concentrations.^51^ Importantly, NT-2Rep is of length similar to that of the LC domains of our artificial model proteins and contains only two residues with a positive solution charge.

During spinning, NT dimerization cross-links spidroins in the nascent fibers and contributes to self-assembly of the LC repeats. However, it is not known how NT dimerization affects the structure of the adjacent LC regions. As the first step, we used AF3 to predict structures for the monomeric and dimeric state of NT-2Rep. The ordered NT domain is confidently predicted with pLDDT scores >70 in the monomeric prediction and >90 in the dimer, indicating high confidence in the accuracy of the predicted model, whereas low scores of <50 in the partially disordered LC repeat domains indicate poor confidence, as expected for a disordered domain (Figure 4b). Despite their low scores, homorepeat structures like the polyalanine sequences of the repeat domains can be predicted with good accuracy.^52^ Furthermore, the pTM score is increased for the dimer over the monomer (Figure S4), indicating further ordering of the total structure, leading to increased confidence. Since the dimer is made up of two identical subunits and the NT domain is a known ordered domain, this increase is likely to be located in the LC repeats.

Despite the increase in the confidence scores, the model for the LC domains remains ambiguous. Since we found that nMS accurately reflects the disorder content of proteins with LC domains, we turned to nMS to complement the predictions. We recorded mass spectra of NT-2Rep^22^ at pH 8 and 6 and compared the resulting CSDs to the AF3 models (Figure 4c). At pH 8, we observe monomers in two distinct CSDs, with the lower charged region of 7+ to 9+ representing protein with a collapsed disordered region and the higher charged region of 10+ to 22+ representing protein with an extended disordered domain. At pH 6, we find that the protein dimerizes, which strongly suggests that the NT domains remain correctly folded during ESI. We observe two widely spaced CSDs for the dimer, the lower CSD ranges from 10+ to 12+ and the higher CSD ranges from 19+ to 29+. All peaks exhibit the previously established charging adduct behavior involving NH_4_^+^ ions to reach their final charge (Figure S4).

To find out why NT-2Rep dimers populate two highly separated CSDs, we considered three possible states: both protomers in the dimer having extended LC domains, both protomers having compact LC domains as predicted by AF3, and lastly, one protomer having an extended LC domain and the other having a compact one. The theoretical average charges as a function of MW were calculated for these states as described above (Figure 2b) and compared to the measured CSDs (Figure 4d). The lower CSD lines up closely with the predicted average charge for two completely compact repeat domains. The higher CSD agrees well with the expected charge for the two extended repeat domains. Interestingly, there are no charge states representing the third option of one extended and one compact repeat domain. This suggests that the LC domains compact via intermolecular interactions. This cooperative compaction matches the alignment of these domains in the predicted structure and suggests that NT dimerization promotes the assembly of the polyalanine regions in the LC repeats.

### CSDs Allow Direct Quantification of Histone Tail Disorder in Nucleosomes

Considering the good quantitative agreement between the fraction of the protein that is disordered and its contribution to the overall charge, we last asked whether similar quantitative agreements can be found in more complex protein systems. To find out, we chose nucleosomes, which are composed of eight histones surrounded by double-stranded DNA. Importantly, the histones have disordered N-terminal tails, 10–40 amino acids in length, that mediate self-assembly of nucleosomes via LLPS.^53^ The tails are rich in positively charged residues that can engage in “fuzzy” interactions with neighboring nucleosomes as well as DNA. In previous reports, IMMS and MD simulations were employed to assign the CSDs to nucleosomes with different histone tail conformations to different conformational states; the lowest CSD represents the nucleosome with all tails collapsed onto the surface, whereas the middle and highest CSDs represent the nucleosome with four and eight extended histone tails, respectively.^54^

**Figure 5:**
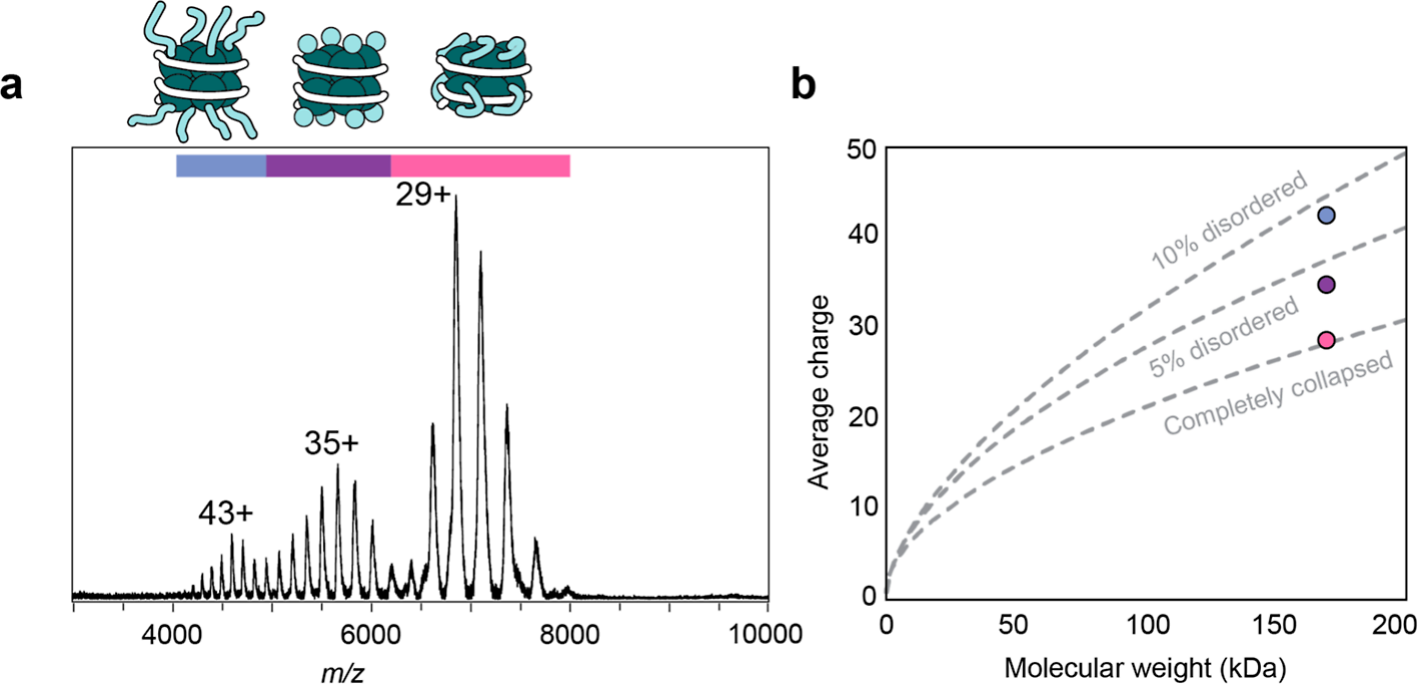
CSDs indicate the extent of disorder in intact nucleosomes. (a) Native mass spectra for nucleosomes show trimodal CSDs. The highest charge state envelope is highlighted in blue and centered on 43+, while the intermediate envelope (purple) and the lowest envelope (pink) are centered on 35+ and 29+, respectively. (b) Empirical calculations for the average charge as a function of molecular weight are shown for a protein that contains 10% disordered and 90% ordered (upper dashed line) or 5% disordered and 95% ordered region of the total molecular weight (middle dashed line) and completely collapsed state (lower dashed line). The average charges of each of the three CDSs are shown using the same color code as in (a).

Based on our observations for NT*-LC proteins, we reasoned that the relative amount of disorder in each population can also be determined directly from the average charges of each population, without interference from the excess of residues with a positive solution charge on the histone tails. To test this hypothesis, we performed nMS of recombinant *Xenopus laevis* nucleosomes composed of two copies each of histones H2A, H2B, H3, and H4 and a 147-base pair dsDNA (Figure 5a) with histone H2A containing mutations in its acidic core region (E61A, E64A, D90A, and E92A).^55^ The total molecular weight of the complex is 198,088 Da, of which 108,451 Da stem from the histones. We observe three distinct CSDs, centered on the 43+, 35+, and 29+ charge states, with the lowest one being the most intense, in line with previous reports.^54^

We then estimated the relative amount of disorder by dividing the molecular weight of the disordered tails by the molecular weight of the intact nucleosome (PDB entry 1KX5), and we found that the extended tails correspond to ca. 10% of the complex. We then plotted the expected average charges as a function of molecular weight for complexes with 10% and 0% disorder and found a good agreement with the highest and lowest CSDs (Figure 5b). Interestingly, the middle CSD can be approximated as being 5% disordered. While we cannot pinpoint whether this population represents a mixture of extended, partially collapsed, and collapsed tails, the CSDs appear to accurately capture the conformational families identified by IMMS and MD simulations. We conclude that nMS charge states can yield information about disorder in complex protein assemblies.

## Conclusions

Our study demonstrates that nMS effectively captures the conformational states of partially disordered proteins with LC domains. We show that the disorder content can be estimated from the CSDs regardless of the amino acid composition of the disordered domain. Interestingly, the effect is independent of ionizable residues, which leads us to speculate that the CSDs of the model proteins are controlled by their solution confirmation(s) regardless of CEM or CRM ionization. With nMS, we can also detect conformational changes induced by domain interactions, as seen in the compaction of the spider silk LC regions upon dimerization. However, it should be noted that not all proteins will display a quantitative agreement between the charge state and disorder observed for our designed model proteins. For example, disordered proteins that engage in intramolecular interactions display low charges and populate compact states in the gas phase.^19^ Furthermore, proteins with highly charged disordered regions may be ejected from the ESI droplet in a way that promotes the unfolding of any folded domains, leading to a more unfolded appearance. While there likely are protein-dependent differences, our findings highlight nMS as a powerful method for exploring the structural dynamics of phase-separating proteins and their role in biological processes.

## Supporting Information Available

Sequences and molecular weights of the designed proteins; materials and methods; CSDs of the chargeless NT*-LC variants following chemical denaturation; charge-state-dependent adduct formation; analysis of NT*-(GSGAP)_14_, NT*-(GSGAY)_14_, and NT*-(GSGAE)_14_ in negative ionization mode; and AF3 confidence scores of NT2Rep monomer and dimer (PDF)
