# Supplementary material for: Native Mass Spectrometry Captures the Conformational Plasticity of Proteins with Low-Complexity Domains
Source: JACS Au. 2025 Jan 8;5(1):281–90. doi: 10.1021/jacsau.4c00961 (PMC11775691; doi:10.1021/jacsau.4c00961)
Supplement: Supplementary file 1 — au4c00961_si_001.pdf [file au4c00961_si_001.pdf]

Supplementary information for

Native mass spectrometry captures the conformational plasticity of proteins with low-complexity domains

Hannah Osterholz, Alexander Stevens, Mia L. Abramsson, Dilraj Lama, Klaus Brackmann, Anna Rising, Arne Elofsson, Erik G. Marklund, Sebastian Deindl, Axel Leppert, Michael Landreh

Correspondence: michael.landreh@icm.uu.se

Document contains:

Supplementary Table 1
Materials and Methods
Supplementary References
Supplementary Figures 1 - 4

Table S1. Sequences and molecular weights of designed proteins

Name	Sequence	Mass after TEV cleavage (Da)
NT*-(GSGAE)$_{14}$	MHTTPWTNPGLAENFMNSFMQGLSSMPG FTASQLDKMSTIAQSMVQSIQSLAAQGRTS PNDLQALNMAFASSMAEIAASEEGGGSLST KTSSIASAMSNAFLQTTGVVNQPFINEITQL VSMFAQAGMNDVSAGSGAEGSGAEGSGA EGSGAEGSGAEGSGAEGSGAEGSGAEGS GAEGSGAEGSGAEGSGAEGSGAEGSGAE ENLYFQSGHHHHHH	20322.77
NT*-(GSGAY)$_{14}$	MHTTPWTNPGLAENFMNSFMQGLSSMPG FTASQLDKMSTIAQSMVQSIQSLAAQGRTS PNDLQALNMAFASSMAEIAASEEGGGSLST KTSSIASAMSNAFLQTTGVVNQPFINEITQL VSMFAQAGMNDVSAGSGAYGSGAYGSGA YGSGAYGSGAYGSGAYGSGAYGSGAYGS GAYGSGAYGSGAYGSGAYGSGAYGSGAY ENLYFQSGHHHHHH	20409.85
NT*-(GSGAP)$_{14}$	MHTTPWTNPGLAENFMNSFMQGLSSMPG FTASQLDKMSTIAQSMVQSIQSLAAQGRTS PNDLQALNMAFASSMAEIAASEEGGGSLST KTSSIASAMSNAFLQTTGVVNQPFINEITQL VSMFAQAGMNDVSAGSGAPGSGAPGSGA PGSGAPGSGAPGSGAPGSGAPGSGAPGS GAPGSGAPGSGAPGSGAPGSGAPGSGAP ENLYFQSGHHHHHH	19961.86
NT*-(GSGAK)$_{14}$	MHTTPWTNPGLAENFMNSFMQGLSSMPG FTASQLDKMSTIAQSMVQSIQSLAAQGRTS PNDLQALNMAFASSMAEIAASEEGGGSLST KTSSIASAMSNAFLQTTGVVNQPFINEITQL VSMFAQAGMNDVSAGSGAKGSGAKGSGA KGSGAKGSGAKGSGAKGSGAKGSGAKGS GAKGSGAKGSGAKGSGAKGSGAKGSGAK ENLYFQSGHHHHHH	20396.67

Materials and Methods

Protein expression and purification

All chemicals were purchased from Sigma unless noted otherwise. Plasmids were ordered from GeneScript. For NT2Rep (*1*), NT*-(GSGAE)$_{14}$, NT*-(GSGAY)$_{14}$, and NT*-(GSGAP)$_{14}$, expression the plasmid was transformed into competent *E. coli* BL21 (DE3) cells. Single colonies were inoculated into 20 mL LB medium with ampicillin (100 µg/mL) and grown overnight (37 ºC, 200 rpm). 500 mL LB medium with ampicillin (100 µg/mL) were inoculated with this at a 1:100 ratio and at OD_{600} = 0.7-0.9 protein expression was induced (0.5 mM IPTG, 4h, 30 ºC, 200 rpm). The same procedure was used for NT*-(GSGAK)$_{14}$ with the difference that carbenicillin (100 µg/mL) was used instead of ampicillin, expression was induced with 1 mM IPTG, and the cells were grown overnight before harvesting.

The cells were harvested by centrifugation (20 min, 5 000 g, 4 ºC) and resuspended in 20 mM Tris, pH 8, with protease inhibitor. After sonication (6 min, 40% amplitude, 2 s on 8 s off) the lysate was centrifuged (20 min, 20 000 g, 4 ºC) and the protein was purified from the filtered (0.22 µm) supernatant by Ni affinity chromatography with 20 mM tris, 500 mM imidazole pH8 buffer. The sample was dialyzed overnight to remove the imidazole, and the His-tag was cleaved by TEV digest overnight. In a final reverse IMAC, TEV and uncleaved protein were removed and the cleaved protein was collected and concentrated.

Recombinant histones (H2A APM, H2B, H3 C110A and H4) from *Xenopus laevis* were purified essentially as described (*2*). Proteins were expressed in BL21 (DE3) pLysS (Novagen). Expression was induced with 1 mM IPTG at OD_{600} = 0.7 for 2 h at 37 °C. The cell pellets were resuspended in 40 mM NaOAc pH 5.2, 1 mM EDTA, 10 mM lysine, 200 mM NaCl, 5 mM β-mercaptoethanol, 6 M urea, and protease inhibitor (Roche). The cells were lysed using a sonicator and the lysate was cleared by centrifugation. The supernatant was filtered through a 0.45 µm filter and purified over a 5 mL SP HP column (GE Healthcare), with a 5 mL Q HP column (GE Healthcare) and eluted with a salt gradient. Peak fractions were dialyzed overnight against cold water. The dialysate was mixed with Tris pH 8.0 to a final concentration of 15 mM and then passed over a 5 mL Q HP column (GE Healthcare). The final protein was concentrated to 5 mg/ml and stored at -80 °C.

Microscopy

Proteins were buffer exchanged into 100 mM ammonium acetate, pH 8, using Zeba Spin desalting columns (Thermo Fisher Scientific) and bright-field microscopy images were acquired using a Nikon Eclipse Ti series inverted microscope (Nikon) equipped with a Crest X-light V2 series confocal unit (Nikon) using a Plan Apo 10x objective (Nikon) and a Zyla sCMOS camera (Andor).

Native mass spectrometry

nESI capillaries were purchased from Thermo and used for all nMS experiments. NT*-LC proteins were buffer exchanged into 100 mM ammonium acetate, pH 8, with an approximate protein concentration of 10 µM. Mass spectra were acquired on a Q Exactive Plus (Thermo Fisher Scientific), modified for high-mass analysis (MS Vision, NL). The capillary voltage was set to 1.5 kV, the source temperature to 30 ºC and the fore vacuum (ion source) was set to 2 mbar. The higher-energy collision dissociation (HCD) voltage was 0 V except for collisional activation experiments (0 V – 40 V). Data was analyzed using Xcalibur 2.2 (Thermo Fisher Scientific). Repeating the measurements on different days and in some cases with protein from different purifications showed high reproducibility.

Intact nucleosomes were buffer-exchanged into 50 mM ammonium acetate, pH 6.9, using Zeba desalting column (Thermo Fisher). Mass spectra were acquired on a Waters Synapt G1 modified for high-mass analysis (MS vision). Capillary voltage was set to 1.5 kV, source pressure maintained at 8 mbar, source temperature 30°C, cone voltage 50 V and trap voltage 50 V. All mass spectra from the Synapt were visualized using MassLynx 4.1 (Waters).

Computational Analysis

LLPS propensity was assessed using the FUzDrop server (https://fuzdrop.bio.unipd.it/predictor) (*3*). Protein structures were predicted using AlphaFold 3 (https://alphafoldserver.com/) (*4*). Representative ensembles of the designed LC domains were generated using IDPGan (https://github.com/feiglab/idpgan) (*5*).

Average charge states for NT-LC proteins were calculated from the spectra shown in Figure 2a. Charge state envelopes were selected according to the color coding: extended 23+ to 12+, intermediate 11+ to 9+, and compact 9+ to 7+. The average charge z(avg) was calculated as follows:

$$z(avg) = (\sum (z(i) \times Int(i))/\sum (Int(i)$$

with z(i) being the charge of each peak in the envelope and Int(i) the corresponding intensity. Formulas for the predicted charges (z_{pred}) of NT*-LC proteins were based on reports by Testa et al. and Kaltashov and Mohimen (*6*, *7*). Confidence intervals for folded proteins are reported by Abramsson et al. (*8*).

For NT*-LC variants, the formulas were:

Compact state:

$$z_{pred} = 0.048 \times MW^{0.5223}$$

Folded NT* with compact LC domain:

$$z_{pred} = 0.048 \times (MW \times 0.75)^{0.5223} + 0.048 \times (MW \times 0.25)^{0.5223}$$

Folded NT* with extended LC domain:

$$z_{pred} = 0.048 \times (MW \times 0.75)^{0.5223} + 0.0141 \times (MW \times 0.25)^{0.7368}$$

Fully extended state:

$$z_{pred} = 0.0141 \times MW^{0.7368}$$

For NT-2Rep dimers (Figure 3d), the formulas were:

Compact state:

$$z_{pred} = 0.048 \times MW^{0.5223}$$

For the NT2Rep dimer with one extended 2Rep tail:

$$z_{pred} = 0.048 \times (MW \times 0.88)^{0.5223} + 0.048 \times (MW \times 0.12)^{0.522}$$

For the NT2Rep dimer with two extended 2Rep tails:

$$z_{pred} = 0.048 \times (MW \times 0.75)^{0.5223} + 0.0141 \times (MW \times 0.25)^{0.7368}$$

For nucleosomes (Figure 4b), the formulas were:

Compact state:

$$z_{pred} = 0.048 \times MW^{0.5223}$$

Compact state with 5% disordered histone tails:

$$z_{pred} = 0.048 \times (MW \times 0.90)^{0.5223} + 0.048 \times (MW \times 0.1)^{0.5223}$$

Compact state with 10% disordered histone tails:

$$z_{pred} = 0.048 \times (MW \times 0.95)^{0.5223} + 0.0141 \times (MW \times 0.05)^{0.7368}$$

Supplementary References

1. N. Kronqvist, M. Otikovs, V. Chmyrov, G. Chen, M. Andersson, K. Nordling, M. Landreh, M. Sarr, H. Jörnvall, S. Wennmalm, J. Widengren, Q. Meng, A. Rising, D. Otzen, S. D. Knight, K. Jaudzems, J. Johansson, Sequential pH-driven dimerization and stabilization of the N-terminal domain enables rapid spider silk formation. *Nat. Commun.* **5**, 3254 (2014).
2. L. C. Lehmann, L. Bacic, G. Hewitt, K. Brackmann, A. Sabantsev, G. Gaullier, S. Pytharopoulou, G. Degliesposti, H. Okkenhaug, S. Tan, A. Costa, J. M. Skehel, S. J. Boulton, S. Deindl, Mechanistic Insights into Regulation of the ALC1 Remodeler by the Nucleosome Acidic Patch. *Cell Rep.* **33**, 108529 (2020).
3. M. Vendruscolo, M. Fuxreiter, Sequence Determinants of the Aggregation of Proteins Within Condensates Generated by Liquid-liquid Phase Separation. *J. Mol. Biol.* **434**, 167201 (2022).
4. J. Abramson, J. Adler, J. Dunger, R. Evans, T. Green, A. Pritzel, O. Ronneberger, L. Willmore, A. J. Ballard, J. Bambrick, S. W. Bodenstein, D. A. Evans, C.-C. Hung, M. O'Neill, D. Reiman, K. Tunyasuvunakool, Z. Wu, A. Žemgulytė, E. Arvaniti, C. Beattie, O. Bertolli, A. Bridgland, A. Cherepanov, M. Congreve, A. I. Cowen-Rivers, A. Cowie, M. Figurnov, F. B. Fuchs, H. Gladman, R. Jain, Y. A. Khan, C. M. R. Low, K. Perlin, A. Potapenko, P. Savy, S. Singh, A. Stecula, A. Thillaisundaram, C. Tong, S. Yakneen, E. D. Zhong, M. Zielinski, A. Žídek, V. Bapst, P. Kohli, M. Jaderberg, D. Hassabis, J. M. Jumper, Accurate structure prediction of biomolecular interactions with AlphaFold 3. *Nature* **630**, 493–500 (2024).
5. G. Janson, G. Valdes-Garcia, L. Heo, M. Feig, Direct generation of protein conformational ensembles via machine learning. *Nat. Commun.* **14**, 774 (2023).
6. L. Testa, S. Brocca, R. Grandori, Charge-Surface Correlation in Electrospray Ionization of Folded and Unfolded Proteins. *Anal. Chem.* **83**, 6459–6463 (2011).
7. I. A. Kaltashov, A. Mohimen, Estimates of Protein Surface Areas in Solution by Electrospray Ionization Mass Spectrometry. *Anal. Chem.* **77**, 5370–5379 (2005).
8. M. L. Abramsson, L. J. Persson, F. Sobott, E. G. Marklund, M. Landreh, Charging of DNA Complexes in Positive-Mode Native Electrospray Ionization Mass Spectrometry. *J. Am. Soc. Mass Spectrom.*, jasms.4c00335 (2024).

Supplementary Figures

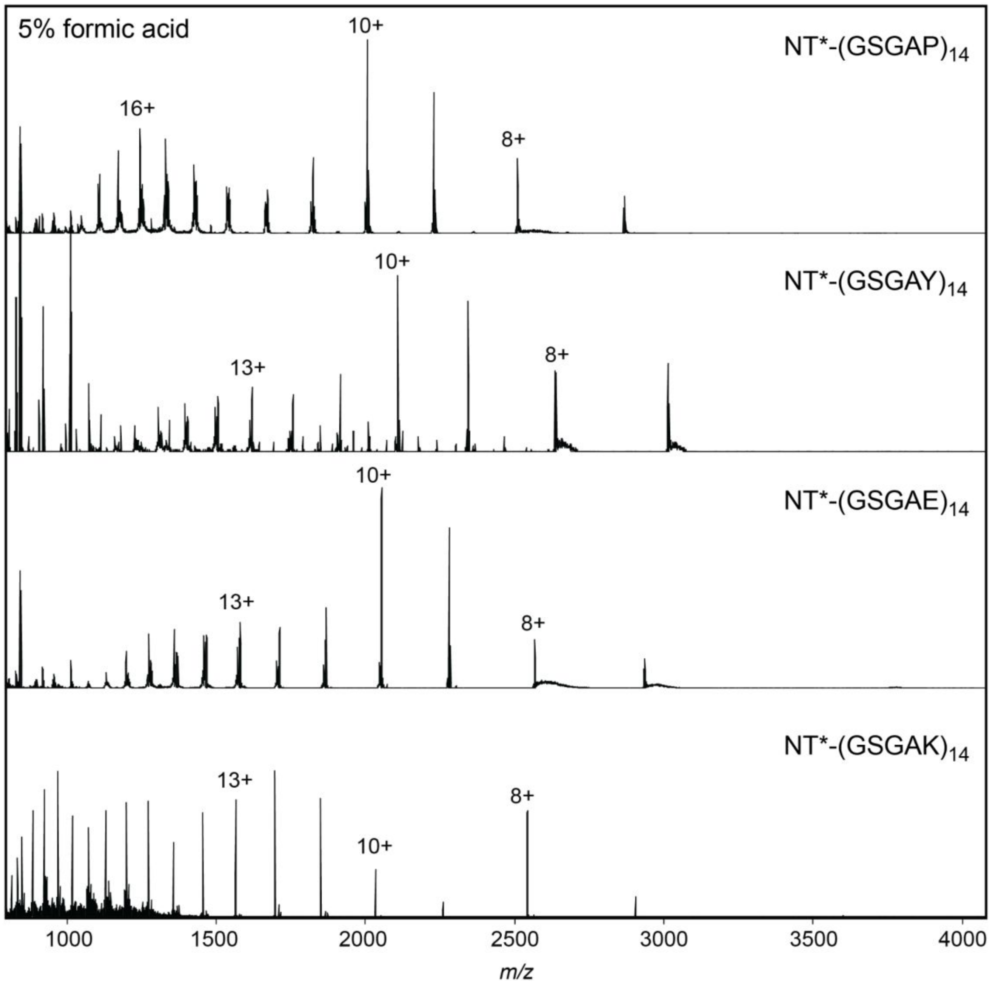

Figure S1: CSDs of the chargeless NT*-LC variants following chemical denaturation. *No significant change for the highest and middle CSDs is observed, while the signal intensity of the lowest CSD around the 8+ charge state is notably reduced. The ammonium adduct pattern for NT*-(GSGAE)$_{14}$,NT*-(GSGAY)$_{14}$ and NT*-(GSGAP)$_{14}$ is the same as in Figures 2 and 3. Denatured NT*-(GSGAK)$_{14}$ (bottom) shows higher charge states than in the native state and no adducts.*

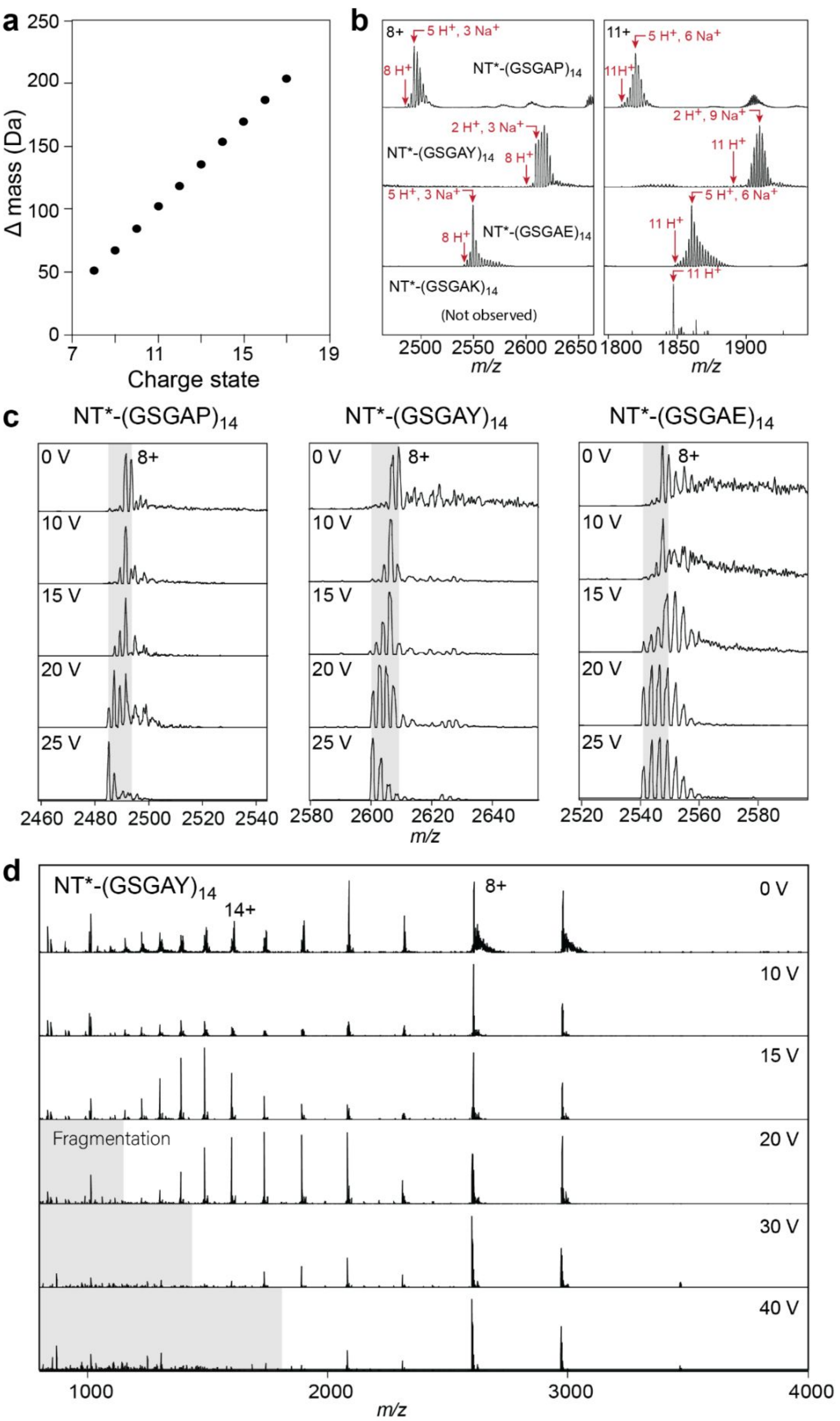

Figure S2: Charge-state dependent adduct formation. (a) *Plot of mass difference (Δ mass) for each charge state of NT*-(GSGAP)$_{14}$ sprayed in 100 mM ammonium acetate shows a linear increase by multiples of 17 Da.* ***(b)*** *Native mass spectra in the absence of ammonium acetate show Na adduct formation that correlates with ion charge. No adducts were observed for NT*-(GSGAK)$_{14}$.* ***(c)*** *Collisional activation of the 8+ ion results in the loss of ammonia adducts and retention of a proton, but requires*

higher trap voltages than for the 14+ charge state (Figure 3b). NT-(GSGAE)$_{14}$ (right) retains more ammonium adducts than NT*-(GSGAP)$_{14}$ or NT*-(GSGAY)$_{14}$, likely due to a more stable association with the negatively charged glutamate sidechains.* ***(d)*** *Activation leads to charge reduction above trap voltages of 15V, which is the onset of fragmentation (grey areas) as shown for NT*-(GSGAY)$_{14}$.*

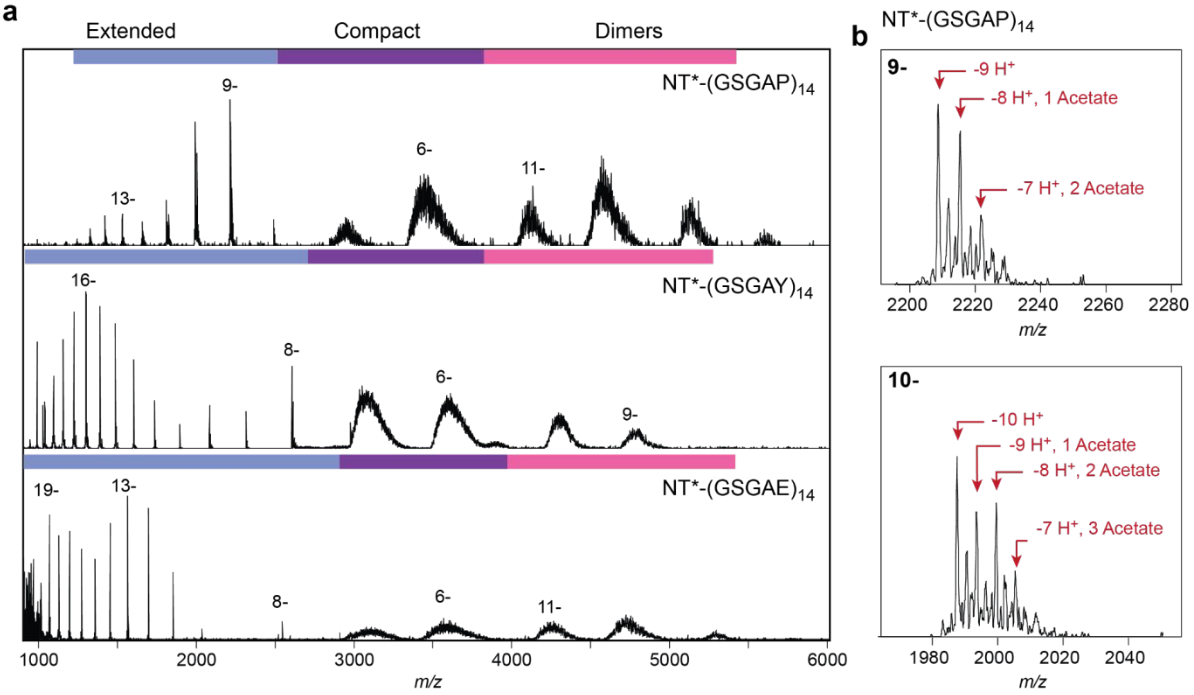

Figure S3: Analysis of NT*-(GSGAP)$_{14}$. NT*-(GSGAY)$_{14}$, and NT*-(GSGAE)$_{14}$ in negative ionization mode. *(a) All three proteins show highly and lowly charged monomers as well as a small dimer population. (b) NT*-(GSGAP)$_{14}$ retains acetate adducts whose total number correlates with ion charge. NT*-(GSGAP)$_{14}$. contains seven sites with negative charge in solution (see Figure 1b).*

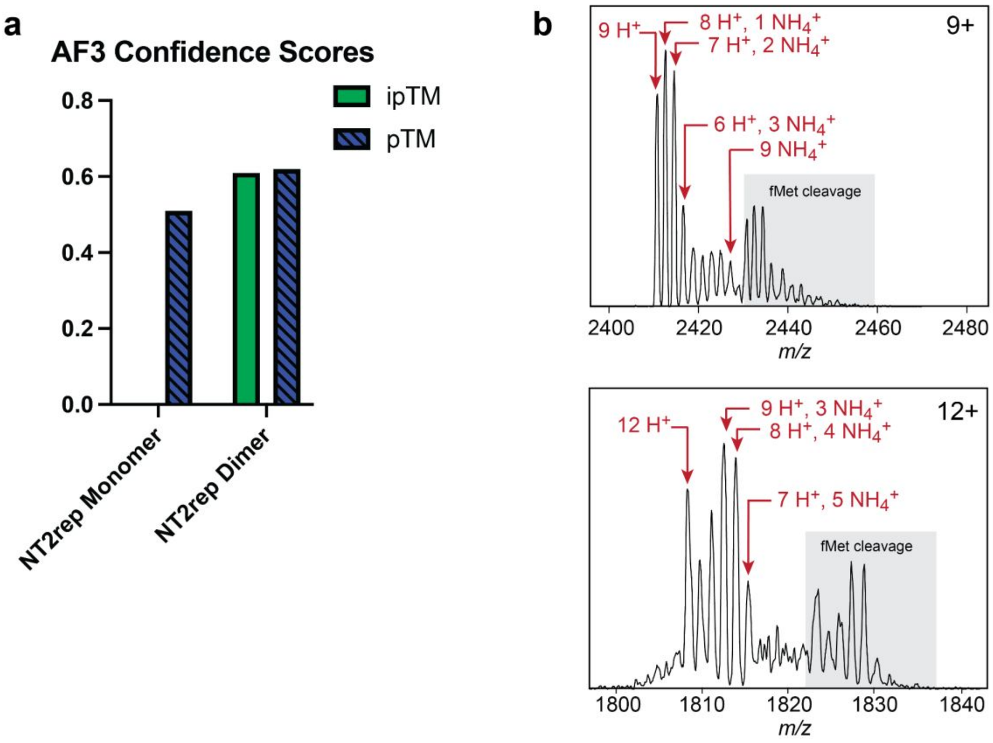

Figure S4. *(a) AF3 confidence scores of NT2Rep monomer and dimer. (b) NT2Rep displays the same adduct pattern as the NT*-LC constructs, with seven positive sites being protonated and ammonium adducts accounting for the additional charges for each charge state.*
